# Engineered 3D Polymer and Hydrogel Microenvironments for Cell Culture Applications

**DOI:** 10.3390/bioengineering6040113

**Published:** 2019-12-13

**Authors:** Daniel Fan, Urs Staufer, Angelo Accardo

**Affiliations:** Department of Precision and Microsystems Engineering, Delft University of Technology, Mekelweg 2, 2628 CD Delft, The Netherlands; D.Fan@tudelft.nl (D.F.); U.Staufer@tudelft.nl (U.S.)

**Keywords:** 3D microenvironment, additive manufacturing, biomaterials, cell culture, tissue engineering, polymer, hydrogel

## Abstract

The realization of biomimetic microenvironments for cell biology applications such as organ-on-chip, in vitro drug screening, and tissue engineering is one of the most fascinating research areas in the field of bioengineering. The continuous evolution of additive manufacturing techniques provides the tools to engineer these architectures at different scales. Moreover, it is now possible to tailor their biomechanical and topological properties while taking inspiration from the characteristics of the extracellular matrix, the three-dimensional scaffold in which cells proliferate, migrate, and differentiate. In such context, there is therefore a continuous quest for synthetic and nature-derived composite materials that must hold biocompatible, biodegradable, bioactive features and also be compatible with the envisioned fabrication strategy. The structure of the current review is intended to provide to both micro-engineers and cell biologists a comparative overview of the characteristics, advantages, and drawbacks of the major 3D printing techniques, the most promising biomaterials candidates, and the trade-offs that must be considered in order to replicate the properties of natural microenvironments.

## 1. Introduction

Human bodies are extremely complex architectures composed of organ systems (e.g., digestive, respiratory, cardiovascular, skeletal, nervous) which in turn are divided into organs (e.g., intestine, lungs, heart, brain) constituted by smaller unit blocks: the tissues and the single cells [1]. For several decades, scientists belonging to different research fields, from engineering to biology, biotechnology, physics, and chemistry, have been trying to develop artificial replica of natural cellular microenvironments. The final aim is to mimic their most important features in terms of topology, porosity, stiffness, and biochemistry. Over the years, we witnessed an evolution from basic petri-dish 2D cell culture models towards more realistic 3D microphysiological systems [2] representing a valid tool for several applications spanning from basic cell biology studies to in vitro drug screening and tissue engineering.

The aim of the present review is twofold. On the one hand it provides to micro-fabrication scientists a set of biology-driven specifications, which are dictated by the properties of the natural microenvironments to be mimicked. On the other hand, it lets cell-biologists discover the opportunities in the realm of 3D fabrication and the limitations of these approaches. In Section 2 we discuss the typical requirements to be fulfilled in terms of stiffness, porosity, biocompatibility, and so on. Section 3 presents different manufacturing techniques. Following an iterative approach, the most important characteristics of the 3D structures that can be achieved with the respective techniques are highlighted. Processing and material aspects are addressed, as well as structural and mechanical properties, and biological characteristics. A similar structure is used for Section 4, however now starting from the material. It is devoted to the description of synthetic and nature-derived materials and how they can be shaped using 3D microfabrication. Section 3 and Section 4 serve as a quick reference guide to relevant information for the reader who is interested in either a specific technique or a particular material. The two tables at the end of the article may be used as a guide to relevant paragraphs. Some repetition of information is unavoidable in this approach, for which we apologize to those who read the entire sections. The review concludes with a final discussion on the indication of future directions to be considered in the field of 3D microphysiological systems. 

## 2. Required Microenvironmental Properties for Cell Culture Applications

### 2.1. Stiffness

The stiffness of a material, and more specifically of a biomaterial, is defined as the rigidity of an object resisting deformation in response to an applied force. This property can be evaluated by measuring several physical parameters, also called moduli: the elastic or Young’s modulus (E), the bulk modulus (K), and the shear modulus (G) [3]. In the context of biological applications, the Young’s modulus is undoubtedly the most used factor to evaluate the characteristic rigidity of biomaterials. Its role is indeed of paramount importance in mechanobiology [4,5], a nascent field whose main target is to unveil how mechanical cues of the surrounding microenvironment influence cellular behavior, namely: migration, proliferation, differentiation processes, and diseases related to these processes. In such context, researchers try to emulate the mechanical properties of the natural extracellular matrix (ECM) whose features may largely vary depending on the origin of the tissue to be mimicked. Tissue elasticity can range from less than 1 kPa (brain, lung, breast), 1–10 kPa (endothelial tissue, muscle), 100 kPa (pre-calcified bone), 1 MPa (cartilage), up to 2–4 GPa (bone) [6,7]. The fine control of mechanical properties of the employed biomaterial provides therefore a crucial tool for guiding the fate of stem cells whose final differentiation can be widely tuned as demonstrated, for instance, in human mesenchymal stem cells (hMSCs), able to differentiate into neural, muscle, or bone lineages after being cultured on polyacrylamide gels of varying stiffness (≈0.1–1, 8–17, or 25–40 kPa respectively) [8], and neural stem cells differentiating into neurons or glial cells in the presence of softer (≈1 kPa) or stiffer (≈10 kPa) collagen/hyaluronic acid matrices respectively [9].

### 2.2. Roughness and Porosity

It is unanimously recognized that nano- and micro-topography has a direct influence on cell adhesion and proliferation [10]. Cells are able to distinguish features with a height down to a few nanometers [11,12] and are able to wrap around sub-micrometric structures [13,14]. The increasing sophistication of nanotechnology allowed studying the influence of physical nanotopography on cell behavior in the past years. One of the first demonstrations of how nanoscale features affect the functionality of osteoblasts in the presence of nanophase ceramics dates back to the stroke of the new millennium [15]. More recently, it has been shown that, by mimicking the nanoroughness of amyloid-β plaques (the main hallmark of Alzheimer’s disease), topology plays a role in the loss of functions in neurons affected by neurodegenerative diseases [16]. In summary, as soon as the employed material is patterned in the form of a grooved surface, nanopillars, nanopits, or gradient topologies imitating the ECM, we can observe immediate effects on cell mechanosensitivity [17]. 

Another key parameter of materials employed for cell culture applications is porosity. Through porosity, the amount of void regions (the pores) over the total volume under consideration is estimated. Such a property is of vital importance especially for tissue engineering as it impacts the perfusion of nutrients and oxygen, as well as the creation of microvasculature networks within the engineered cellular microenvironments. Often, pristine materials are not equipped with the level of porosity required in tissue engineering or drug-screening applications. This may lead to the creation of a non-efficient artificial ECM that prevents the desired cell proliferation, differentiation, and migration mechanisms. To address this problem and guide a fine tuning of the porosity, several fabrication and characterization techniques can be employed involving, among other things, salt leaching, freeze-drying, stereolithography (SLA), scanning electron microscopy, and micro-computed tomography imaging [18].

### 2.3. Biocompatibility and Biodegradability

The choice of a material for cell culture applications is dictated by its ability to interact efficiently with cells without inducing cytotoxic effects, i.e., without inducing mortality or unwanted aberrations in their development. The biocompatibility of materials usually employed in microfabrication processes can be a major hurdle especially when dealing with tissue engineering applications and, depending on the type of tissue to be replaced, only some specific scaffolds can be employed to support regeneration and engraftment [19,20]. The origin of the biomaterials, synthetic or natural, plays then a crucial role as it may affect in very diverse ways the immune system of the host. In the microfabrication panorama, techniques such as fused deposition modeling, bioprinting, or electrospinning can count on a wide range of available biomaterials which often can be obtained by exploiting “decellularization” techniques [21]. Such techniques, starting from native tissues/organs, lead to the creation of matrices containing only ECM components. Nevertheless, the resolution of features fabricated with such techniques cannot reach the precision of light-assisted 3D additive manufacturing (such as two photon polymerization (TPP) or SLA) that, on the other hand, show some limitations due to the toxicity of the feedstock materials since most of the required photosensitive polymers are not formulated for biological applications [22]. In such context, the advent of cross-linkable hydrogels opened new possibilities for a more careful tuning of the photoinitiator molecules required to make the material sensitive to light exposure and cell-friendly at the same time [23].

Sometimes biocompatibility is not sufficient, and it is desired that the scaffold is also biodegradable; for instance, when the engineered tissue is implanted together with the scaffold in patients. Once the cultured cellular network has integrated in its natural surroundings, the scaffold should fade away without leaving toxic products. Examples are the treatment of osteomyelitis [24], or targeted drug delivery achieved by hydrogel-based scaffolds that decompose upon exposure to specific environmental conditions like pH or temperature [25].

### 2.4. Biochemical Functionalization

Often, pristine hydrogel or polymeric materials are not immediately suitable for culturing cells. An emblematic case is the one of poly(ethylene glycol) diacrylate (PEGDA), a biocompatible, FDA-approved hydrogel, that by itself cannot provide an ideal environment to support cell adhesion due to its bio-inert nature [25]. The same applies to other polymeric materials, such as poly(methyl methacrylate) (PMMA), that only after being exposed to plasma ion roughening modifications exhibit surface chemistry and morphology changes that promote cellular adhesion [13,26]. It is therefore of paramount importance that the targeted biomaterial can be grafted with components of the natural ECM such as laminin, fibronectin, vitronectin, RGD-peptide, or growth factors [27] by using surface or bulk bio-chemical modification [28]. In such context, it is important to keep in mind that bulk modification is often more desirable as the bioactive molecules are embedded both on and within the biomaterials avoiding the risk of fading completely away in presence of a biodegradable scaffold.

## 3. Techniques for Fabricating 3D Polymer and Hydrogel Cellular Microenvironments

An ideal microphysiological system is a complex 3D structure with micro and macro features, tunable degradation and stiffness, and strong bioactivity able to reproduce the requirements listed in Section 2. Traditionally, porous biocompatible scaffolds have been made without precise control over such variables via techniques such as leaching [29], gas foaming, and electrospinning [30], or gel self-assembly processes [31]. The rise of a range of additive manufacturing (AM) techniques has changed this, allowing fabrication down to 50 nm resolution in the case of light assisted AM, to multi-material fabrication via extrusion, or to the inkjet printing of tailored biocomposites. 

### 3.1. Powder Bed Fusion: Selective Laser Sintering and Binder Jetting

#### 3.1.1. Process and Materials

Selective laser sintering and binder jetting are both techniques that work layer-by-layer and locally join powder grains at the surface of a powder bed. In selective laser sintering (SLS), a layer of powder is spread over a stage and the required bonds are formed by fusing the powder together at desired places via local heating with a high intensity laser [32]. Similarly, binder jetting deposits a binder material to fuse the powder at the desired location. In both cases the stage is lowered after a local fusing step and the next powder layer is spread on top for sintering or binding. 

The base material must be in powder form, and is thus restricted to thermoplastics, ceramics, metals, and oxides. Depending on the powder’s particle size, the micro-porosity, roughness, and mechanical properties can be tuned [33]. However, control over powder formation with precise particle shape and size is difficult without using toxic solvents, and hence mechanical milling is generally used at the expense of loss of control over powder geometry [34].

#### 3.1.2. Structural and Mechanical Properties

Because of the layer-by-layer process, the built 3D structure can be fabricated with overhangs due to the support of the surrounding powder bed during fabrication (although with certain design limits, e.g., totally encapsulated cavities cannot be emptied), and be tailored with stiffness and porosity gradients [35]. Feature and pore sizes can be controlled down to ~350–500 µm for polycaprolactone (PCL) powders [36,37]. Another benefit is that fused powders have a microstructure roughness that leads to better cell adhesion [35].

Powder-bed-produced structures have similar moduli, strength, and brittleness as the constituent particles. For example the mechanical properties of SLS PCL powder is suitable for bone tissue engineering [38,39,40] with compressive modulus of 10–60 MPa and compressive strength of 0.6–10 MPa. These values can be tuned by adapting the laser power and powder particle size [41].

#### 3.1.3. Biocompatibility, Biodegradability, and Bioactivity

Many of the biocompatible thermoplastics and bio-ceramics suitable for powder bed fusion are biodegradable and resorbable in the human body, with tunable degradation times (see Section 4 for further details). One clear benefit of SLS is the absence of additives for crosslinking, such as photo-initiators in the case of photo-polymerization that can be cytotoxic.

One of the main disadvantages of SLS on the other hand is the high local temperature needed for sintering. Even if cells, proteins, and other bioactive molecules are encapsulated within thermoplastic particles during that process, the high temperatures can destroy these bioactive components. To overcome this, surface SLS was used on a combination of polylactic acid (PLA) powder and carbon microparticles by employing infrared (IR) radiation that is well adsorbed in the carbon particles. Since PLA, on the other hand, does not absorb IR, only the surface of the PLA particles are fused due to local heating via nearby carbon black. This opens the possibility of incorporating bioactive species into the PLA, which could survive the thermal treatment [42].

### 3.2. Fused Deposition Modeling or Fused Filament Fabrication

#### 3.2.1. Process and Materials

In fused deposition modeling (FDM) filamentous material is molten and extruded through a nozzle and deposited at specific x-y-z locations to form a freeform 3D structure. The nozzle, chamber, and build-plate are all heated and temperature controlled. Fused filament fabrication (FFF) is the same process except for the fact that only parts of the assembly are temperature controlled. Because of the wide availability and low cost of this type of 3D printer, FDM/FFF is frequently used to create cell-culturing scaffolds. Multiple-nozzle systems have been used to combine different stock materials into one structure or to tune the amount of cross-linker for graded structures. Similarly, in situ UV curing has been added to FDM nozzles for hybrid fabrication systems that deposit filaments and photo-cure them at the same time.

Highly viscous materials such as thermoplastics are suitable to be fashioned into filaments and subsequently deposited via FDM or FFF. The base materials are deposited by first melting the filament to increase flow, and then placing it via a nozzle. Benefits include the high versatility to blend different materials into the filament. On the other hand, the filament needs to be molten, hence heated, which may destroy seeded bioactive materials. 

#### 3.2.2. Structural and Mechanical Properties

The 3D structures are built-up as a freeform. Therefore, the technique does not support fabrication of overhangs. Such structures can be obtained, though, by using sacrificial support structures, which can be mechanically or chemically removed after fabrication. 

The resolution of the structures is restricted by the nozzle diameter. The size of the nozzle is limited by clogging that also depends on the rheological properties of the filament. Typical resolution for FDM is ~100 µm. Deposited thermoplastics features are much smoother than the ones obtained, for example, by SLS. If desired, roughness can be introduced by incorporating nanoparticles in the filament or by plasma reactive ion etching.

The preparation of the filament can significantly affect the mechanical properties of the fabricated structure [43]. This includes incorporation of nanoparticles, copolymerization of the stock filament and in situ curing. Further, the writing direction, raster angle, and layer thickness can affect different mechanical properties of the structure through the thermal gradient during the fabrication process. 

#### 3.2.3. Biocompatibility, Biodegradability, and Bioactivity

The thermoplastics used in FDM are generally biocompatible, and in most of the cases also biodegradable. The degree of cross-linking significantly affects the degradation rate. FDM allows tuning the cross-linking via multiple nozzles, temperature control, and in situ UV curing. As mentioned earlier for SLS, encapsulated bioactive compounds may not survive the high temperature extrusion process in FDM. Two approaches can be taken: loading or coating an already produced FDM scaffold with a bioactive material, or ensuring that the processing temperature and/or extrusion pressure is within limits for survival of encapsulated bioactive molecules.

### 3.3. Extrusion Bioprinting

#### 3.3.1. Process and Materials

Extrusion bioprinting involves the deposition of biomolecules and cells that are encapsulated inside a flowable hydrogel matrix, resulting in a highly bioactive structure. Optimization of printing speed, pressure, and temperature is required for any minute changes in the bioink, such as the concentration of encapsulated cells [44]. Bioinks loaded with polysaccharides and proteins (e.g., collagen, alginate) can show very different values of viscosity. The technology is mature with available bioprinters on the market at reasonable costs, which allow customization and focus on bioink mixtures [45,46,47,48,49,50,51,52,53]. 

#### 3.3.2. Structural and Mechanical Properties

In order to encapsulate cells within the hydrogel matrix it is of paramount importance to optimize the viscoelastic properties of the bioink during extrusion and the mechanical stability of the structure after extrusion. The degree of crosslinking, method of crosslinking, and other processing parameters can affect mechanical properties, biodegradability, cytotoxicity, and bioactivity. To improve shear properties during extrusion, techniques such as the inclusion of nanoparticles, e.g., nanoclays and polymer nanoparticles, can modulate the rheological properties of the ink. Immediate cross-linking by UV, thermal, or chemical treatment can result in higher print fidelity and structurally stiffer objects. Multiple-nozzle extrusion systems have been realized to allow the 3D printing of bioactive structures with good mechanical integrity via a support structure [54]. The typical feature size of bioprinted structures are ~500 µm.

#### 3.3.3. Biocompatibility, Biodegradability, and Bioactivity

In general, bioprinted materials based on polysaccharides and proteins are already highly bioactive. Methods for tuning the biodegradation rate include: hybridization of the biopolymer, controlling the degree of crosslinking, application of oxidation, and mixing with different support materials such as sodium citrate while avoiding the use of cytotoxic solvents and cross-linkers. To increase bioactivity, specific cells can be encapsulated within the bioink or coated during post-processing. Encapsulated cells undergoing extrusion bioprinting can have greatly varying bioactivity based on the extrusion pressure, shear stress, and nozzle size. Therefore, process optimization needs to be performed not only for facilitating bioink flow and mechanical stability during and after writing, but also for biodegradability and bioactivity.

### 3.4. Inkjet Printing

Inkjet printing is a manufacturing technique where the bioinks usually hold low viscosity. They form droplets, which are ejected by pressure pulses that are either generated with piezoelectric elements, or via rapid heating and subsequent volume expansion. Similar challenges exist as with extrusion bioprinting apply, namely cell damage during and after printing, stability of the printed structures, and print fidelity and resolution. Recent review of inkjet printing and related bioinks can be found here [55,56,57,58].

### 3.5. Light-Assisted Additive Manufacturing

#### 3.5.1. Process and Materials

Photo-structuring methods use light to form 3D constructs via chemical reactions such as crosslinking or bond cleavage. Stereolithography (SLA) employs a laser beam to photo-polymerize a series of transverse-plane image slices of a photo-resin following a layer-by-layer process that finally leads to the realization of a 3D design. It is a high throughput method, and because the technology is mature, cost of production is low. Simplified SLA units exploiting Blu-ray technology have been used to fabricate poly(propylene fumarate)/diethyl fumarate (PPF/DEF) 3D structures with pore size of about 200 µm [59]. Digital light processing (DLP) follows the same layer-by-layer mechanism of SLA but each 2D layer of the 3D design is exposed in one shot by using UV light sources. Two-photon polymerization (TPP) uses the non-linearity of two-photon absorption to polymerize extremely confined regions (voxels have a characteristic dimension in the order of 100 nm) of resin since only the focal spot receives sufficient light intensity for triggering the two-photon process. TPP has much higher 3D resolution than SLA but the writing time is much slower, making it suitable for special applications and rapid prototyping but not for high throughput production [60,61,62,63], although the integration of galvanometric mirrors and moving-beam fixed-sample (MBFS) strategies are starting to fill this gap.

Base materials used for light-assisted manufacturing must be photo-crosslinkable, which is often achieved by mixing them with a photoinitiator. Many biocompatible polymers can be hybridized and made photocurable by using the technique of ring-copolymerization. 

#### 3.5.2. Structural and Mechanical Properties

Photo-polymerization methods produce the highest resolution features (below 10 µm). The process allows the creation of true 3D features such as overhangs without the need for sacrificial supports (contrary to extrusion based strategies). TPP in particular allows the fabrication of very high resolution (below 1 µm) freeform 3D shapes in photocurable materials. The mechanical properties can be tuned not only by the degree of photo-polymerization (e.g., controlled through the exposure dose or the concentration of photoinitiator), but also by the synthesis of the monomer or prepolymer (e.g., copolymer mixtures, molecular weights, other additives). 

#### 3.5.3. Biocompatibility, Biodegradability, and Bioactivity

The downside of light-assisted manufacturing is that the type of useable photo-polymers are limited and the use of solvents and photoinitiators may leave behind cytotoxic residues which may cause biochemical damage to cellular networks. Recent research has focused on discovering less cytotoxic photoinitiators in combination with more biocompatible, compliant, gel-like materials [33,64]. Printing using visible light mediated by chromophores avoids the use of harmful UV light that can damage encapsulated cells. Light can also be used as a heat source for curing, thereby avoiding the use of photoinitiators. Much like extrusion bioprinting, varying the concentration of additive materials allows the tuning of biocompatibility and biodegradability.

### 3.6. Hybrid Methods

Combinations of the above manufacturing techniques can be used for the formation of interesting composites. For example, the extrusion of a PCL mesh followed by inkjet printing of keratinocytes was used to print a model of human skin [65]. Extrusion of rigid PCL was combined with SLA printing of soft poly(ethylene glycol) diacrylate (PEGDA) to quickly build composite scaffolds with pore sizes of about 350 µm in diameter, improving the hydrogel rigidity as well as viability of cells [66]. Yet another example of a hybrid method to form hierarchical structures is to use FDM combined with gas foaming: a poly(vinyl alcohol) (PVA)/PLA blend was deposited via FDM, followed by gas foaming to form micropores of ~10 µm diameter. The PVA was subsequently removed by dissolving it in a solvent, forming macropores of 100–800 µm diameter [67]. Alternatively, hierarchical structures were fabricated by using a PEG–PCL blend and 3D printing a scaffold to form the macrostructure. Micropores were then formed on the surface by chemical treatment, increasing the hydrophilicity [68]. In addition, SLA can be combined with electrospinning, to form 3D constructs composed of PCL fibers with hydrogel patterns. These soft/rigid combinations offer both excellent biocompatibility and mechanical strength, which can be exploited for culturing neural stem cells [69].

### 3.7. Replication

Indirect techniques or replication techniques use pattern transfer to avoid direct fabrication disadvantages such as thermal heating or the use of cytotoxic materials. However, not all shapes and dimensions can be transferred. For example, replication via an intermediate PDMS mold allows features as small as 500 nm to be transferred from photo-resin into PLA. This results in high-resolution, albeit 2.5D, topographical features which are nevertheless useful in studying the effect of topographical cues in cell culturing [70]. In another recent work [71], a silicone-elastomer/hydrogel interpenetrating network was fabricated by 3D printing PVA filament into a scaffold, pattern transfer into PDMS, and pattern transfer again into silicone-poly(2-hydroxyethyl methacrylate)-*co*-poly(ethylene glycol) methyl ether acrylate (pHEMA-*co*-PEGMEA), providing excellent mechanical properties and controlled drug release of the elastomer combined with the biocompatibility and hydrophilicity of the hydrogel.

## 4. Synthetic and Natural Biomaterials for Building Cell-Instructive Microphysiological Environments

The choice of materials not only depends on the required features for cell culture applications as detailed in Section 2, but also depends on the processing requirements of that material as described in Section 3. By combining, mixing, and hybridizing different materials with vastly different properties, specific functions such as biocompatibility, biodegradability, mechanical stiffness, structural strength, cytotoxicity, and bioactivity can be tuned. In such context we can mainly discriminate between synthetic and naturally derived biomaterials. The first ones (Table 1) are highly reproducible and can be easily integrated within microfabrication strategies but often lack physiological features. On the other hand, the second ones (Table 2), which are often originating from the natural extracellular matrix, hold properties very close to the natural tissue but their extraction can be laborious and lead to significant batch-to-batch variability.

### 4.1. Thermoplastics

Synthetic thermoplastic polymers such as polycaprolactone (PCL), poly propylene fumarate (PPF), polylactic acid (PLA), polyglycolic acid (PGA), and their co-polymers, have mechanical properties well suited for use as scaffold structures in the regeneration of cells for bone and cartilage tissue engineering, as they are relatively stiff and strong. Because of their thermoplasticity and the ability to be modified via ring co-polymerization, they are suitable for a wide variety of additive manufacturing techniques. They are biodegradable and resorbable thanks to hydrolysis and enzymatic digestion over a tunable period of up to ~2 years which is in the range for bone healing. They are biocompatible and non-toxic, however they have poor hydrophilicity and lack bioactivity [33,72,73,74,75].

#### 4.1.1. Polycaprolactone (PCL)

##### Process and Material

PCL is a Food and Drug Administration (FDA) approved biocompatible polymer [38,41,76] with a low melting temperature (~60 °C) suitable for almost all of the main additive manufacturing techniques [77]. PCL structures have been fabricated via SLS [38,39,40], FDM [78,79], and extrusion bioprinting [80,81]. Further, PCL can be functionalized with acrylate groups and mixed with a photoinitiator to enable photopolymerization [82].

##### Structure, Feature Size, and Porosity

PCL structures fabricated via SLS can achieve a porosity of 40–80% [41], while those fabricated via FDM can hold a porosity of 44–78% [79]. FDM fabricated feature sizes of ~160 µm were reported [79], while bioprinted PCL structures with feature sizes of ~200 µm were produced highlighting how process parameters can affect the morphology of the extruded PCL [80,81,83]. Concerning light-assisted fabrication, PCL structures with resolution down to 50 µm via DLP has been achieved [84]. TPP was used to decrease the feature size even further, down to 1 µm for 3D structures [85]. By tuning the number of synthesized acrylate groups, decreasing the molecular weight of the oligomers, and varying the concentration of acrylated PCL, structures with ~10 µm pore size, 3 µm feature size, and decreased polymerization threshold were produced via TPP [86]. Methacrylated copolymers of PCL and PLA were also used via TPP to produce scaffolds of ~300 µm pore size [87].

##### Mechanical Properties

SLS fabricated PCL structures were found to have mechanical properties suitable for bone and cartilage tissue engineering, with tunable compressive modulus (10–60 MPa) and compressive strength (0.6–10 MPa) [38,39,40,41]. Tuning parameters include laser power, powder size, and the presence of additives. Among these additives, bio-ceramics such as tricalcium phosphate (β-TCP) [36,88,89,90] and hydroxyapatite (HA) [91,92,93] have been most commonly studied. These composites allow stiffness tuning, including the incorporation of stiffness gradients [94]. By adding 30% β-TCP to 70% PCL, the compressive modulus was increased from 6.77 MPa to 13.66 MPa [36]. The compressive modulus and strength were both lowered with the inclusion of HA, from ~1.8 MPa to ~1.2 MPa and ~0.4 MPa to ~0.2 MPa respectively [92].

FDM fabricated PCL structures with tunable tensile modulus (4–77 MPa) and tensile strength (0.4–3.6 MPa) have been reported [79]. FDM is particularly flexible in terms of additives and composites. Using PCL as a base the following mixtures with a large variation in mechanical properties were synthesized: bioactive glass (compressive modulus increase of ~40% up to ~150 MPa [95]); soft elastomers (Young’s modulus reduction to ~750 kPa, tensile strength reduction to ~300 kPa, and maximum elongation of 57% [96]); polyurethane formulations (tensile strength of 8–21 MPa and maximum elongation of 200–720% [97]); bio-ceramics such as β-TCP (a 33% increase in Young’s modulus and yield strength [90]); polymers such as PLA [98,99] (tensile strength of 45 MPa and 5.5% elongation using TiO_2_ as filler [100]), and PPF [93]; and micro-crystalline cellulose (compressive modulus between 7–32 MPa, flexural modulus of 55–76 MPa, yield strength 4–6.9 MPa [101]).

Bioprinted structures made of a mixture of PCL and polyethylene glycol dimethyl-acrylate (PEGDMA), achieved a suitable viscosity for production and showed an elastic modulus of ~65 MPa and hardness of ~6 MPa when processed in a nitrogen environment [83].

The mechanical behavior of structures fabricated by photopolymerization can be adapted by changing the molecular weight and functionality of the prepolymers. For example, low molecular weight (300 g/mol) prepolymer of PCL triol leads to a higher tensile modulus of 6.9 MPa and a low strain of 13% at break [84,86] compared to the high molecular weight (1250 g/mol) PCL diol, which leads to a tensile modulus of 3.3 MPa and 39% strain at break. Also, the ratio of polymers can be reformed to control mechanical properties. For TPP of methylacrylated copolymer of PCL with PLA, ratios of PLA:PCL of 16:4, 18:2, and 9:1 resulted in compressive moduli of 0.27, 2, and 4 MPa respectively. Similarly, the tensile modulus and strength increased as the PCL content decreased [87].

##### Biocompatibility and Biodegradability

Because of the low melting temperature of PCL, solvents can be avoided preventing cytotoxicity [82]. PCL scaffolds produced by FDM show good biocompatibility, as demonstrated by fibroblast and osteoblast proliferation [78]. For blended materials, PCL membranes with incorporated β-TCP particles were shown to have better biocompatibility than standard collagen membranes [102], while PCL blended with graphene and carbon nanotubes (CNT) were shown to have decreased cytotoxicity exhibited by significant cell affinity after 28 days [103,104], although in the case of CNTs the cytotoxicity depends on the physiochemical properties of the CNT [103].

The biodegradation of PCL has been shown to be tunable over periods of up to 2 years. Higher molecular weight prepolymers and prepolymers with high chemical functionality both decrease the biodegradation time. The functionality and subsequent degree of cross-linking has a more significant effect on biodegradation. Up to 2.5 times slower degradation has been observed for highly cross-linked PCL compared to loosely cross-linked structures [84]. Similarly, the polymer ratio PLA/PCL was found to affect biodegradation rates [87].

##### Bioactivity

PCL showed improved or tailored bioactivity upon incorporating the following substances: poly(3-hydroxybutyrate-*co*-3-hydroxyvalerate) PHBV [105], methylated collagen [106], decellularized bone matrix [107], hydrogels [108], nano-HA [93], poly(lactic-*co*-glycolic acid) PLGA [90], hyaluronic acid [109], or TiO_2_ [100]. Graphene/PCL blends have been investigated for extrusion printing because of graphene’s electroactive nature, improving its bioactivity and hydrophilicity [103,104]. Chitosan hydrogels have been injected into PCL scaffolds for bone tissue engineering applications [110]. Piston extruded HA/PCL blends show good bioactivity, attributed to the increased surface roughness [111]. Separately, other HA/PCL scaffolds have been chemically treated by alkaline erosion to expose the HA crystals within the scaffold to improve bioactivity [112]. Such composites enhance PCL biocompatibility as proved by increased cell proliferation, distribution, and differentiation [90,92,113,114,115,116,117] as well as by the ability to target specific bone tissue engineering outcomes. In vivo tests, however, are still inconclusive [116].

PCL scaffolds can alternatively be post-coated with bioactive materials. The incorporation of hyaluronic acid and gelatin based hydrogel has been shown to not only increase bioactivity and production of glycosaminoglycan, but also to improve cyto-compatibility [37]. PCL scaffolds coated with collagen seeded with chondrocytes show increased cell proliferation [118], depending on the geometry of the scaffold. PCL scaffolds fabricated by SLA have been coated with poly-dopamine to enhance osteogenesis and angiogenesis of hMSCs, desirable for bone tissue engineering [119].

#### 4.1.2. Polypropylene Fumarate (PPF)

##### Process and Material

Reviews by Cai [120] and Diez-Pascual [121] reported information on the synthesis of PPF, its properties, 3D manufacturing techniques, and applications. Salt leaching, gas foaming and electrospinning are the most common techniques used for fabricating PPF scaffolds, while top-down methods are rare. There have only been few recent reports of fabrication of PPF via FDM [122] and none via SLS. The main top-down 3D fabrication method has been SLA and DLP. PPF has the benefit of a low curing temperature of ~54.7 °C [123].

##### Structure, Feature Size, and Porosity

PPF mixed with the DEF/TCP and photoinitiator BaPO have been used to fabricate scaffolds via micro-stereolithography (µ-SLA), with pore sizes down to 150 µm and porosity up to ~80% [124]. Because PPF is highly viscous, dilution in a solvent such as DEF is necessary for control over feature size [125]. Besides substrates for tissue engineering and cell culturing, microneedles with a tip radius of ~50 µm for drug delivery have been fabricated using PPF and µ-SLA [126].

##### Mechanical Properties

PPF can be modified by additives in ways similar to PCL, i.e., with bioceramics such as TCP (compressive modulus of 146–161 MPa, compressive strength of 109–133 MPa [123]) and HA (compressive modulus 50 MPa for 10 wt% of HA in PPF [127]); oxides such as TiO_2_ (flexural modulus of 1 GPa, flexural strength of 42 MPa, Young’s modulus of 230–580 MPa [128]); or CNTs (flexural modulus of 0.75–1 GPa, flexural strength of 15–27 MPa, compressive modulus of 1.3–2 GPa, compressive strength of 53–81 MPa [129]) all used as reinforcement materials. Other ways to tune mechanical strength and stiffness are copolymerization, or variation of the molecular mass, or crosslinking density [120,130]. For example, increasing the molecular mass from 1500 Da to 2450 Da reduced the compressive stiffness from 27 MPa to 17 MPa. PPF/DEF/BaPO could be polymerized using a 308 nm XeCl laser enabling tuning of the stiffness between 4 MPa to 4 GPa simply by varying the laser intensity and repetition rate [131]. The length of post-curing can also affect mechanical properties [132] and can lead to an increase of the compressive modulus up to 2.5 times [59]. Specifically, increasing post-curing energy from 110 J to 1100 J increased the elastic modulus from 1 MPa to 9 MPa [132].

##### Biocompatibility and Biodegradability

PPF degrades in the body into a Krebs cycle constituent (fumaric acid) and a food additive (propylene glycol). In addition, the degradation properties of PPF structures can be tuned by the use of ring-opening copolymerization methods to synthesize PPF with specific molecular masses; the integration of diluents such as DEF to change its viscosity; and the tuning of photoinitiators’ concentration to affect the polymerization kinetics [125,133].

##### Bioactivity

There are a number of ways to increase bioactivity of PPF structures. Copolymerization with PEG has been one popular approach [134], while surface functionalization with peptides also improves cell attachment, allowing applications in drug delivery and cell culturing [120]. Other functional coatings include arginine–glycine–aspartate peptides to support proliferation of human chondrocytes for cartilage tissue engineering [124,135], as well as PPF scaffolds loaded with collagen and coated with neurotrophin-3 to promote bioactivity and specifically the growth and proliferation of neurons and axons [136]. Apart from offering structural support and pores for neuronal regrowth, the stiff PPF scaffold also provided mechanical cues guiding cells to grow in specific directions [136].

Besides coating, materials can be mixed into the feedstock: Au nanoparticles (NPs) were incorporated into PPF/DEF scaffolds [137] showing greater adipose stem cell adhesion in vitro than without the Au NPs, while in vivo tests showed absence of immune response in animals, offering a significant step towards tissue regeneration [138]. Hydroxyapatite NPs have similarly been incorporated into PPF membranes which allow controlled release of the NPs as the film degrades [139].

Finally, in vitro studies of rat bone-marrow stromal cells show that osteogenic signal expression of these cells are enhanced depending on the pore size and ratio of DEF content [140]. Human MSC differentiation down to the three mesenchymal lineages was supported, with the shape of the pores having an effect [132]. For in vivo studies, PPF scaffold pore size and porosity did not seem to affect tissue response, although degradation and reduction in inflammatory cells was observed [141].

#### 4.1.3. Polylactic Acid (PLA)

##### Process and Material

PLA is a thermoplastic widely used for 3D additive manufacturing. It is adaptable and has been used together with SLS [34,142], FDM [143,144,145,146,147,148,149], and light-assisted techniques [61,150]. For light-assisted fabrication, photopolymerizable PLA can be synthesized by methacrylation and mixed with a photo-initiator to form light active photoresin. A review of PLA for additive manufacturing for biomedical applications is given by van den Eynde [151].

##### Structural and Mechanical Properties

Using FDM, PLA scaffolds have been fabricated with pore sizes of ~150 µm and line-widths of ~100 µm [143], while for SLA, PLA scaffolds with pore sizes of ~1 mm have been achieved [150]. For TPP features with 20 µm size were printed [61].

PLA has the highest mechanical strength of all biodegradable polymers, with a tensile modulus of 3–4 GPa, tensile strength of 50–70 MPa, flexural modulus of 4–5 GPa, and flexural strength of 100 MPa [34], suitable for bone tissue engineering applications [152]. It is however brittle with low impact toughness and an elongation at break of 2–10% [34]. This can be improved and tuned by blending PLA with other materials such as PCL, PGA, and polyurethane for stiffening and hardening [153]. Concerning SLS of PLA powders, PLA was combined with calcium carbonate powder to lower bending strength to 75 MPa [34]. On the other hand in FDM of PLA, materials such as nano-hydroxyapatite (nHA) [147,148] and graphene oxide (GO) [147,149] can be mixed into the filament to enhance mechanical properties. For example Kothapalli reported the compressive modulus of PLA scaffolds to increase from 4.7 to 9.8 MPa upon the inclusion of 50 wt% nHA while the compressive strength increased from 0.29 to 0.44 MPa [154], albeit using a salt-leaching method for preparation of scaffold. For GO, a tensile modulus of 16.73 MPa and tensile strength of 0.57 MPa for PLA with 15% nHA and 2% GO has been reported [147]. In general, pore size was shown not to affect tensile strength [143].

##### Biocompatibility, Biodegradability, and Bioactivity

PLA is a biocompatible, biodegradable, and non-toxic polyester, but it is limited by its hydrophobicity [152] and lack of bioactivity. For example, in vitro tests with human fetal osteoblasts displayed no cytotoxic effects or changes in biocompatibility after FDM. However, there was reduced growth on PLA scaffolds, possibly due to residual monomers [145]. Similarly, cell culture evaluation using human bone-marrow stromal cells showed not only an absence of cytotoxicity but also a slight increase in cell activity and viability [143]. Further, TPP fabricated PLA scaffolds were shown to be neurocompatible and able to support the growth of Schwann cells [61].

To improve bioactivity, PLA has been blended with PEG, hyaluronic acid, and chitosan [153], as well as GO to improve cell growth and attachment [147,149] and nHA for promotion of osteoconductivity [147,148]. Alternatively, nHA has been used together with PLA to decrease the adherence and proliferation of *Staphylococcus aureus* and *Pseudomonas aeruginosa* bacterial cell colonies [142]. PLA scaffolds produced by FDM can be used as a support for biocomposite materials, such as gelatin–forsterite fibers via electrospinning [146].

#### 4.1.4. Polyether-Ether-Ketone (PEEK)

##### Process and Material

PEEK is a semi-crystalline thermoplastic with high chemical resistance. Production costs are high compared with other thermoplastics, and in addition PEEK has a relatively high wear rate and high melting temperature of ~343 °C, making it difficult to process [155,156]. 3D PEEK structures can be manufactured using SLS, FDM, and extrusion bioprinting. FDM was used, for example, to fabricate PEEK structures which underwent mechanical testing to determine optimum printing parameters [157]. For extrusion based methods, care must be taken with trapped micro-bubbles and temperature management of the head/nozzle, chamber, build-plate, etc., which can affect the mechanical properties and crystallinity of the produced structure [155,158]. Lastly, epoxy functionalized PEEK formulated as a bioink, together with fenchone, was extruded at room temperature and then cross-linked at 380 °C, avoiding thermal stresses during the initial fabrication process [159].

##### Structural and Mechanical Properties

PEEK has a Young’s modulus of ~3.6 GPa and tensile strength of ~100 MPa, making it suitable for bone, dental, and spinal implants [156]. PEEK structures can be optimized during FDM in terms of tensile, compressive, and flexural strength as well as fracture toughness [160,161]. The following processing parameters were used: 1) the direction of writing and subsequently the thermal gradient during the build (elastic modulus of 2.7 GPa and tensile strength of 48 MPa at 360 °C nozzle temperature, and elastic modulus of 4.1 GPa and tensile strength of 84 MPa at 200 °C ambient temperature [158]); 2) the raster angle (0° raster giving tensile modulus of 2.5 GPa and tensile strength of 22.9 MPa while 90° raster giving tensile modulus of 2.06 GPa and tensile strength of 13.4 MPa [159]); 3) the layer thickness (200, 300, 400 µm layer thicknesses giving tensile strengths of 40, 56.6, and 32.4 MPa and compressive strengths of 53.6, 60.9, and 54.1 MPa respectively [161]).

##### Biocompatibility, Biodegradability, and Bioactivity

PEEK is non-toxic [156] but biologically inert [155] with a long biodegradation time [162]. To control degradation rates, PEEK has been blended with other polymers such as PGA (percentage weight loss after 28 days of 10.57% for 20% PGA, 12.88% for 40% PGA, 8.64% without nano-TiO_2_, and 9.72% with nano-TiO_2_ [163,164]) and poly-L-lactide (PLLA) (up to 14% weight loss over 28 days for 50 wt% PLLA [165]) although further studies on its degradation products and their bio-absorbability are required. SLS was used to fabricate scaffolds in both cases, with incorporated nano-TiO_2_ particles for an anti-bacterial function and β-TCP particles for bioactivity and biodegradability, respectively. Further, surface modification of SLS fabricated PEEK scaffolds can be undertaken, for example via impregnation with mesenchymal stem cells [166], resulting in higher osteodifferentiation of bone-derived stem cells.

### 4.2. Soft Polymers

#### 4.2.1. Hydrogels

Hydrogels are very highly hydrated polymer networks, which allow cells to attach, differentiate, and proliferate. A number of reviews have been published in the last decade regarding the additive manufacturing of 3D hydrogel structures used for cell culturing and tissue engineering [167,168,169,170]. Hydrogel gradient scaffolds are very useful in mimicking real biological structures. Extrusion bioprinting [170] and SLA [171] are the two main techniques for producing such complex multi-material structures [172]. Cell-laden hydrogels are typically printed via extrusion bioprinting because the high temperatures involved in sintering and photo-polymerization required for light-assisted fabrication can damage encapsulated cells [167]. The fabrication trade-off for extrusion printed hydrogels is mainly between shape fidelity and structural stiffness versus bioactivity.

#### 4.2.2. Polyethylene Glycol (PEG)

##### Process and Material

PEG is a very hydrophilic, biocompatible, and biodegradable polymer with low stiffness in the kPa range. Acrylate terminated PEG such as PEG-methacrylate (PEGMA) and PEG-dithiothreitol (PEGDTT) allow crosslinking, and therefore both extrusion bioprinting and light-assisted fabrication are suitable for tailoring 3D PEG based structures [173,174]. For example, by adding nanosilicates to PEGDTT, shear-thinning properties are tuned to allow 3D printing of PEG hydrogel as a bioink [172]. Concerning light-assisted methods, both PEGMA [173,175] and PEG-diacrylate (PEGDA) [176] when mixed with acid-cleavable crosslinkers and photoinitiators can be used to fabricate 3D PEG scaffolds.

##### Structural and Mechanical Properties

Regarding extrusion bioprinting, PEG hydrogel was combined with sodium alginate and a nanoclay achieving resolutions of ~500 µm [174]. For SLA fabricated PEGMA scaffolds, pore sizes of ~25 µm have been reported [173], while TPP was used [177,178] together with Irgacure photoinitiator to produce PEGMA scaffolds with 5 µm features [179]. Photoprinting of PEG-tetraacrylate (PEG4A) has also been reported, where silk fibroin with melanin nanoparticles were mixed with the PEG4A to increase light absorption and thereby improve resolution, achieving photo-printed PEG4A at ~500 µm resolution [180].

PEG has variable low stiffness in the kPa range depending on the specific molecular weight of the PEG chains. To further tune the stiffness, PEG composites have been synthesized. For example, scaffolds composed of PEG plus sodium alginate resulted in a 3D interpenetrating network with a fracture energy of 1500 J/m^2^ which is higher than the value of articular cartilage [174]. Similarly, scaffolds fabricated via DLP and composed of PEG4A with silk fibroin and melanin nanoparticles resulted in a higher elastic modulus [180] with a storage modulus increase from 1 to 2.5 kPa and a loss modulus increase from 200 to 700 Pa. Copolymerizing PEG with PPF and lithium acylphosphinate as a cytocompatible photoinitiator resulted in a continuous-DLP printed scaffold at ~100 µm resolution with a 10-fold increase in elongation-at-break compared to PPF scaffolds alone [134].

##### Biocompatibility, Biodegradability, and Bioactivity

PEG is biocompatible and biodegradable [134,180]. Modification of crosslinkers used to crosslink acrylate terminated PEG can introduce degradation profiles, for example including 3 wt% of cyclooctyne-hyaluronic acid results in complete degradation in 46 days in pH 9, or 10 days in pH 7.4 with hyaluronidase (HAse) [181]. Concurrently, a study on degradation was reported using rhodamine B mixed with PEGMA as a model drug and its release kinetics shown to be influenced by different device designs, material densities, and porosities [179]. Here, scaffolds with 5 µm features showed slower drug release than scaffolds with 10 µm to 15 µm features, while there was a 37% weight loss after 144 days for the scaffold with 5 µm features and 43% weight loss for the scaffold with 10 µm features. Finally, 3D printed PEGDA scaffolds hosting PEG norbornene (PEGNB) microspheres showed good cytocompatibility and tunable degradation by the addition of PLA–PEG–PLA [182]. Scaffolds with 10 wt% PEGNB showed 6% cell release after 7 days while scaffolds with 5 wt% PEGPLA/NB showed 18% cell release.

Although PEG does not encourage cell adhesion, it represents a very good candidate for the encapsulation of other bioactive materials, drugs, and chemicals. Recently it has been reported how a combination of S-nitroso-N-acetyl-D-penicillamine and PEG–PCL was coated on a 3D printed PLA scaffold for the controlled release of nitric oxide and the realization of antibacterial and blood-compatible vascular grafts [183]. Similarly, PEG norbornene microspheres encapsulated cells for controlled cell delivery and release [182], while curcumin, an antioxidant and anti-inflammatory, was encapsulated in PCL–PEG–PLGA which together was loaded into a hydroxyapatite matrix [184].

An alternative to encapsulation is hybridization of PEG with natural molecules such as chitosan [173], sodium alginate [174], arginine-glycine-aspartic acid (RGD) groups; or copolymerization with peptide diacrylates [25], which improves cell adhesion. Chitosan has been mixed with PEGDA–PCL–diacrylate copolymer, which increased hydrophilicity [185]. Extruded PEG/sodium alginate scaffolds resulted in cultured hMSCs having high viability over 7 days [174].

#### 4.2.3. Polydimethylsiloxane (PDMS)

##### Process and Material

PDMS is an inert, viscoelastic, and non-toxic silicone. It is hydrophobic, and surface treatments such as plasma oxidation or functionalization is needed for hydrophilicity [186,187,188]. As such, in terms of microfabrication for biomedical applications, it is mainly used as an intermediate stamp for pattern transfer. In particular, it is used as a stamp for micro-contact printing of cells and biomolecules onto specific locations of a substrate [189,190,191,192]. High resolution chitosan–gelatin scaffolds have also been casted via an intermediate PDMS mold which itself was replicated from a SLA fabricated master [193].

##### Structural and Mechanical Properties

Combined with in situ UV curing, bioprinting of drug-loaded PDMS via semi-solid extrusion at room temperature was accomplished, with feature sizes of ~250 µm [194]. PDMS-methacrylate macromers have been developed for SLA also with feature sizes of ~250 µm [195]. For high resolution 3D printing of PDMS, TPP was used to produce 3D structures with submicron resolution [196] by mixing photocurable PDMS with a photoinitiator. Besides TPP, a femtosecond laser could be used for local curing of PDMS within a 3D volume showing single feature resolution of ~5 µm and absence of potentially cytotoxic photoinitiators [197]. Finally, TPP has been used with a urethane acrylate resin to produce submicron sized structures which were transferred to PDMS via a molding process [198]. 

Regarding mechanical properties, a PDMS/thiol based photopolymer was developed for SLA with printed structures showing high elongation at break of up to 138%, and Young’s modulus of 0.4–1.7 MPa [199]. Radially gradient pore distributions in scaffolds with pore sizes of 1 mm showed higher elastic modulus with an increase from 52 to 1038 kPa and higher fluid permeability [200] compared to scaffolds without pore gradients. Young’s modulus can be also tuned from 48 to 1783 kPa by varying the ratio of prepolymer to curing agent from 50:1 to 10:1 respectively [201].

##### Biocompatibility, Biodegradability, and Bioactivity

SLA fabricated PDMS scaffolds were shown to support cell viability, although the extraction of solvent used during processing is critical in lowering cytotoxicity [195]. Taking advantage of its hydrophobicity, oil-infused PDMS with encapsulated Ag nanoparticles was 3D printed as an anti-bacterial wound dressing [202].

### 4.3. Polysaccharides

Polysaccharides are naturally occurring sugar chains which are bioactive and can be used as base material for additive manufacturing [203]. In particular, sulfated polysaccharides such as cellulose, chitosan, alginate, carrageenan, heparin sulfate, agarose, etc., typically have inherent bioactivity, allowing them to bind with many cell receptors and growth factors [204]. The degree of covalent or ionic bonding in the polysaccharide is related to the degree of crosslinking, hence to the mechanical properties of the material [205].

#### 4.3.1. Hyaluronic Acid

##### Process and Material

Hyaluronic acid is a glycosaminoglycan which is part of the extracellular matrix and has a role in cell proliferation and migration [206]. Hyaluronic acid can be crosslinked to form hydrogels capable of being printed into 3D structures, as well as mixed into bioinks or used to functionalize surfaces. Modifications of hyaluronic acid can help to improve the viscoelastic and rheological properties during extrusion as well as the stability after extrusion-based fabrication. For example hyaluronic acid has been modified with hydrazide and aldehyde groups allowing shear thinning and self-healing of the bioink due to formation of physical crosslinks mediated by hydrazine bonds [207]; crosslinked with Pluronic F-127 and dopamine conjugated gelatin to introduce Herschel–Bulkley rheological properties and improve thermal gelling [208]; and combined with methacrylated collagen [209] for improving the printing process.

##### Structural and Mechanical Properties

For extrusion based printing, hyaluronic acid has been grafted with hydroxyethyl acetate and gelatin-methacrylate to form a hydrogel which was 3D printed into a scaffold with ~500 µm resolution [210]. One way to tune mechanical robustness and macro-porosity beyond that of hydrogels is to formulate hyaluronic acid cryogels (hydrogels produced through controlled freezing and thawing of a polymer solution), which have also been 3D printed [211]. In this case the addition of 0–1% w/v of PEG4A varied the Young’s modulus between 2–2.5 kPa, pore size between 100–30 µm, and porosity between 77–70%, respectively. To further improve the stability of hyaluronic acid based composites after extrusion, photo-crosslinkable hyaluronic acid-based hydrogels have been developed [212]. For example, methacrylated hyaluronic acid (MeHA) could be crosslinked with UV light after extrusion to improve mechanical properties [213]. For the addition of 1–3% w/v MeHA, before UV light the storage modulus was 5–200 Pa respectively, while after UV light the storage modulus was 170–2602 Pa respectively, and an overall Young’s modulus after UV light of 1.3–10.6 kPa respectively. Such developments has led to photopolymerizable hyaluronic acid-based bioinks mixed with chondrocyte cells for SLA 3D printing at feature sizes of ~300 µm [214]. A combined hyaluronic acid/poly-DL-lactic acid/PEG polymer system was printed via projection SLA to achieve structures with compressive modulus of ~780 kPa, suitable for cartilage tissue engineering [215].

##### Biocompatibility, Biodegradability, and Bioactivity

Hyaluronic acid and modified hyaluronic acid show good cyto- and bio-compatibility [207,210], as it is naturally a part of the extra-cellular matrix. Extruded hyaluronic acid structures show excellent bioactivity, with hyaluronic acid based hydrogel scaffolds for improving cell viability [208], promotion of stromal cell elongation with applications in building liver models for drug screening [209], retinal cells culturing [216], immobilization of peptides for mesenchymal stem cell culturing with high angiogenic and osteogenic activity [217], deposition of regenerative scaffolds directly during surgery [218], and supporting human adipose progenitor cell and stromal cell adhesion and proliferation [211]. Hyaluronic acid combined with alginate and fibrin has been bioprinted for peripheral nerve tissue regeneration, showing good Schwann cell elongation [219].

Light-assisted crosslinking of hyaluronic acid structures has also showed good primary cell survival and osteogenic differentiation [213]. Tyramine-modified hyaluronic acid involving photo-crosslinking with visible light avoids the relatively high intensity radiation of UV light which might be a concern for cell viability [220]. Finally, a hyaluronic acid/poly-DL-lactic acid/PEG biodegradable photopolymer was used to encapsulate human adipose-derived stem cells and printed via SLA, achieving live cell 3D constructs and resulting in high cell viability [215].

#### 4.3.2. Chitosan

##### Process and Material

Chitosan is a linear aminated polysaccharide derived from chitin, the exoskeletons of shellfish. Functionality is determined by the degree of acetylation and molecular weight, affecting solubility, mechanical strength, biodegradability, and bioactivity [221]. Chitosan scaffolds have been bioprinted via extrusion using silk particles as a filler material to improve the flow properties of the hydrogel ink, the print accuracy, mechanical reinforcement, surface roughness, and biological performance [222,223]. A thorough investigation into the processing parameters of bioprinting using chitosan based bioinks such as their rheological properties and solvent evaporation can be found here [224]. Also SLA has been used to print chitosan 3D scaffolds, whilst both laser induced µ-SLA and TPP have been used on chitosan-g-oligolactide copolymers where the molecular weight of chitosan and oligolactide chains affected the fabrication process of these hydrogels [225,226]. Finally, hybrid manufacturing techniques such as the use of SLA to produce the required 3D structure in a photoresin and microreplication by casting of chitosan/gelatin on PDMS molds, allow the resulting chitosan/gelatin layers to be stacked layer-by-layer to produce multilevel hierarchical structures [227].

##### Structural and Mechanical Properties

Extrusion bioprinted chitosan scaffolds with feature sizes of ~50 µm have been reported [224]. Ear-shape scaffolds with pore sizes of ~50 µm have been printed by SLA from a chitosan/PEGDA composite. By controlling the molecular weight of chitosan and ratio of chitosan, PEGDA, and photoinitiator, the printability, mechanical strength, and cell adhesion could be tuned [173]. The addition of chitosan into PEGDA increases the viscosity of the resin, and the minimum required PEGDA for printability dropped from 30 to 6.5% w/v. For feed ratios of low molecular weight chitosan to PEGDA of 1:5 to 1:10, the swelling reduced from 8.4 to 8.1% respectively, while increasing the ratio from 1:5 to 1:15 increased Young’s modulus 7-fold. For changes in the photoinitiator concentration between 0.025 to 0.03%, the Young’s modulus was 160–680 kPa respectively [173]. DLP was used together with a chitosan/PCL/PEGDA resin to fabricate scaffolds with feature sizes of ~100 µm [185]. Chitosan/allyl bromide produced via a solvent free process was used for TPP at a resolution of 400 nm [228]. Besides the various fabrication techniques, chitosan can be combined with bioceramic hydroxyapatite to print hollow 3D scaffolds for improved vascularization and mechanical properties appropriate for bone tissue engineering [229].

##### Biocompatibility, Biodegradability, and Bioactivity

Chitosan as a biopolymer promotes a variety of bioactivity such as being antioxidant, anti-inflammatory, anti-microbial, or able to trigger homeostasis [230,231]. Concerning light-assisted fabrication of chitosan/PCL/PEGDA composite, tuning the concentration of chitosan allows the composite scaffold to be made more biocompatible [185], where 10 and 15% chitosan specimens showed significantly higher cell viability compared to 0 and 5% chitosan. Other composites include chitosan/polyelectrolyte gelatin for wound dressings [232] and skin tissue engineering [231], chitosan/agarose/alginate with induced pluripotent stem cells for human neural tissue engineering [233], hydroxybutyl chitosan and oxidized chondroitin sulphate for articular cartilage tissue engineering [234], and chitosan/sodium alginate hydrogel as part of asymmetric membranes used as skin constructs [235].

#### 4.3.3. Alginate

##### Process and Material

Alginate is a biocompatible, naturally occurring polysaccharide with highly reactive cross-linking ability which can be further improved with calcium ions as crosslinking promoter, making them suitable not only for extrusion printing but also inkjet printing as shape can be retained quickly [236]. For extrusion, hydrogels need to be prepared such that the viscoelastic properties and shear modulus during extrusion is appropriate for proper printing of features at high fidelity and resolution. Approaches include tuning the degree of crosslinking of the bioink and formation of composites or mixtures such as with NiCu NPs [237], e-polylysine [238], carrageenan [239], gelatin [240,241,242], and nanocellulose [243,244]. Rheological studies show composition ratio, printing temperature, extrusion pressure, and crosslinking concentration affect fidelity and resolution [239,240,242,245]. In comparison to other materials, there has been little work on light assisted 3D printing of alginate. Using visible light, hydrogelation was induced in a mixture of alginate, tris(bipyridine)ruthenium(II) chloride and sodium persulfate. Visible light not only has less toxic and adverse effects on encapsulated cells, but also has deeper penetration depth for thicker constructs [246,247].

##### Structural and Mechanical Properties

For extrusion bioprinting, an alginate/gelatin composite was printed at a resolution of ~150 µm with a Young’s modulus of 280 kPa [240]. For hydrogelation using visible light, print feature size was ~500 µm [246], while the addition of CaSO_4_ increased feature size to ~600 µm [242]. The print resolution can be tuned via oxidation of alginate [248], while controlling the degree of crosslinking allows tuning of the mechanical properties [248]. For example Naghieh et al. showed 3 mL of 50 mM CaCl_2_ crosslinker increased the elastic modulus from 40 to 273 kPa after 1 day of crosslinking and that CaCl_2_ controls the viscosity of the bioink [249]. Concerning alginate/gelatin mixtures at a ratio of 1–4% alginate, the pore sizes were 300–610 µm, and the compressive modulus from 2–6 kPa respectively [242]. In addition, to improve mechanical stability, multi-nozzle systems have been used to simultaneously print a support structure made of PCL alongside alginate hydrogel structures [250,251]. Other ways to improve the mechanical stability after print are by formulation of alginate ink mixed with PVA powder (printability decreased and compressive modulus decreased from 56 to 13 kPa with increasing PVA content from 0 to 30% v/v [252]), or TiO_2_ (Young’s modulus of 13 MPa compared with control of 9 MPa), or β-TCP (Young’s modulus of 8 MPa) [253]. For light-assisted fabrication, methacrylated alginate hydrogels have been photopolymerized with UV light allowing tunable swelling and mechanical properties [254].

##### Biocompatibility, Biodegradability, and Bioactivity

Alginate does not interact directly with mammalian cells, normally is not degradable and typically needs to be modified to induce cell activity [255]. However, in ionically crosslinked configuration, the release of the divalent ions can disrupt the network of alginate and crosslink the gel to the surrounding matrix [256]. Degradation can be tuned by controlling the degree of crosslinking [248,254], the amount of oxidation [248], and by mixing with sodium citrate [257]. For example the dry weight of partially oxidized alginate changed from an initial 20 mg to 0 mg after 9 days in physiological buffer solution [258]. To achieve bioactivity, alginate based hydrogels are typically mixed with bioactive molecules, e.g. via the encapsulation of cells, including myoblasts [242], endothelial cells [259], *E. coli* bacteria [260], transforming growth factor [250], human adipose derived stem cells [248], human induced pluripotent stem cells [261], and human chondrocytes [243,244,251]. In all the above cases, elastic modulus decreased over time while cell activity increased, showing cell viability despite the encapsulated cells undergoing the extrusion process. Alginate hydrogels encapsulating stem cells have been cryogenically preserved to allow 3D bioprinting at any time [262]. For structures fabricated via light-assisted methods, encapsulated chondrocytes have been reported to be viable [263] but without any particular advantage in proliferation or matrix production [264]. This might be related to the non-degradability of methacrylated alginate whose surface morphology is not beneficial to bioactivity [264]. Finally, returning to extrusion based fabrication, another method for producing bioactive alginate scaffolds is via peptide modification, where composites of alginates conjugated with different peptides showed superior Schwann cell viability and directional neurite outgrowth [265].

#### 4.3.4. Cellulose

##### Process and Material

Cellulose is a linear polysaccharide found in many plants and bacteria. It is the most abundant organic polymer on Earth and is thus cost-effective, sustainable and biocompatible. Cellulose can be found as nano-crystals, nanofibers, or in bacterial form, where they can have high surface area. Nano-cellulose crystals and fibers have been widely mixed with alginate to form bioinks which have excellent shear-thinning properties allowing improvement in printability as well as fast cross-linking to achieve shape fidelity [244,266,267,268,269,270,271,272]. For light-assisted printing, cellulose has been mainly used as a filler and reinforcement material to stiffen and strengthen hydrogels and soft polymers. PEGDA structures reinforced with nano-cellulose crystals have been printed via SLA [273,274] with good shape fidelity, mechanical strength (tensile strength increase from 0.6 to 1.2 MPa with the addition of 0.3 wt% of nano-cellulose), and surface wettability [273]. There have been a number of recent reviews specifically focused on the use of nano-cellulose for additive manufacturing of scaffolds in tissue engineering and cell culturing [275,276,277,278,279].

##### Structural and Mechanical Properties

Hydroxypropyl cellulose has itself been hybridized with methylacrylic anhydride to allow photo-crosslinking. The resulting structures produced via photolithography had pore sizes of ~50 µm with a print resolution of ~500 µm [280]. Post-processing of SLA fabricated PEGDA/nano-cellulose by freeze drying resulted in aerogel scaffolds with porosity up to 90% [274].

Cellulose nanofibers can self-assemble and aggregate into fibrils with high elastic modulus and tensile strength. Nano-crystalline cellulose has a tensile strength of ~7.6 GPa and an elastic modulus of 110–220 GPa in the axial direction and 10–50 GPa in the transverse direction, making them excellent reinforcement material for bioinks [276]. For example, galactoglucomannan methacrylates with different concentrations of nano-cellulose allowed tuning of Young’s modulus between 2.5–22.5 kPa [281]. Besides bioinks, nano-cellulose crystals coated with lignin have been used as filler in methacrylate resin and printed with SLA, improving thermal stability and mechanical properties [282]. The tensile modulus was tuned from 0.67 to 0.63 GPa with the inclusion of 1% lignin coated nano-cellulose crystals. Apart from being used as a filler, cellulose itself can be printed, and it was reported that by using *N*-methylmorpholine-*N*-oxide as a solvent, cellulose printed at different temperatures resulted in structures with a range of stiffness from rigid to gel-like [283].

##### Biocompatibility, Biodegradability, and Bioactivity

Cellulose is biocompatible, and its surface can be easily modified to be bioactive, but care must be taken to avoid cytotoxicity. One disadvantage is the absence of natural degradation pathways for cellulose in the human body, and therefore it is still mainly used as an additive. However, it was reported that hydroxypropyl cellulose hybridized with methylacrylic anhydride showed fast biodegradability via hydrolysis together with non-cytotoxicity [280].

The encapsulation of chondrocytes for cartilage tissue engineering [244,269,271], human derived induced pluripotent stem cells [266], human bone marrow derived mesenchymal stem cells [272], pancreatic cancer cells [268], and fibroblast and hepatoma cells [270], all showed increased bioactivity such as cell expression, proliferation, and viability. However, a more detailed study of printing process parameters show that optimum pressure, shear stress, and nozzle size can greatly affect the bioactivity of the encapsulated cells, i.e., for nozzle diameters below 400 µm, cell morphology and proliferation suffered, while for nozzle diameters below 200 µm cell viability also was affected [267].

Besides encapsulation, mixing of cellulose with other bioactive materials is commonly used. The alginate/nano-cellulose bioink system, including variations such as sodium alginate [268], alginate sulphate [267], and the various phases of nano-cellulose, have been reported to have better performance than, for example, hyaluronic acid/nano-cellulose [266]. Gelatin/nano-cellulose based bioinks combined with oxidation also have no cytotoxicity, a pore size of ~600 µm, good cell viability, improved mechanical properties (compressive modulus from 0.5 to 8.5 kPa with the addition of 2% w/v nano-cellulose), and improved print properties [284,285,286]. PEGDA/nano-cellulose structures fabricated by SLA showed high NIH 3T3 cell viability [274]. 

### 4.4. Proteins

Proteins and peptides are highly bioactive molecules which perform specific biological functions, many of them are part of the natural extracellular matrix, and they are widely employed in bioprinting applications [45,287].

#### 4.4.1. Collagen

##### Process and Material

Collagen is a structural protein that is the main component of connective tissue within the human body. As a bioink, collagen is difficult to keep in liquid form. Temperature and pH control during the extrusion process is required. Collagen has also low viscosity and is slow to polymerize, making it difficult to print. One solution is to print collagen within another hydrogel matrix as support, called freeform reversible embedding of suspended hydrogels [288]. For light-assisted manufacturing of collagen structures, a PEGDA/collagen composite hydrogel was used to print a meniscus shape via SLA [289]. Further to this, indirect methods were used such as coating a TPP fabricated acrylate based scaffold with collagen [290], or loading collagen into a ceramic scaffold followed by gel casting [291]. A thorough review of using collagen as a biomaterial for cell culturing can be found here [292] and more specifically for collagen based bioinks, here [293].

##### Structural and Mechanical Properties

Depending on the degree of mineralization, several types of collagen with different mechanical properties can form, varying from that found in bone, cartilage, skin, hair and reticulate fibers. Collagen hydrogels produced for 3D bioprinting are typically rather compliant, although it was shown that compressive modulus could be improved from 10 to 30 kPa with higher collagen concentrations up to 17.5 mg/mL [294]. To further promote crosslinking and thereby improve mechanical properties, the cross-linking agent genipin was used. Pore sizes of ~400 µm and an increase in compressive modulus from 17 kPa to 1.4 MPa (5 mM genipin after 48 h crosslinking) was achieved [295]. For the indirect method of coating collagen onto a TPP fabricated scaffold, ~30 µm pores were reported [290].

##### Biocompatibility, Biodegradability, and Bioactivity

Collagen scaffolds fabricated via heat extrusion showed good shape fidelity and cell viability over ten days [294]. Highly crosslinked collagen fabricated using the crosslinking agent genipin allowed osteoblast-like cells and human adipose stem cells to be viable and to proliferate [295]. Tissue spheroids encapsulated in collagen [296], keratinocytes and fibroblasts encapsulated in collagen [297], as well as primary mesenchymal stem cells [298], were all bioprinted for applications in thyroid gland engineering, human skin engineering, and human meniscus engineering, respectively.

Collagen has also been used as part of mixed polymer systems such as collagen/alginate/gelatin for the proliferation of human corneal epithelial cells [257]; collagen with sodium alginate for the proliferation and gene expression of chondrocytes for cartilage tissue engineering [299]; collagen microfibers within a gelatin methacrylate (GelMA) matrix as well as collagen/agarose for the viability, spreading, and differentiation into osteocytes of bone mesenchymal stem cells [300,301,302]; and collagen/hyaluronic acid for 3D liver microenvironments containing primary human hepatocytes and liver stellate cells [209].

#### 4.4.2. Fibrin

##### Process, Material, and Structural Properties

Fibrin is a fibrous protein involved in blood clotting. Although it can form the base for various bioinks, fibrin, like collagen, is difficult to print because of the low viscosity of its precursor fibrinogen solution. It is typically mixed with other materials to improve printability, such as gelatin, alginate, or genipin. By extruding fibrinogen mixed with hyaluronic acid and PVA into thrombin solution, fibrin crosslinking was improved and scaffolds with ~100 µm features were printed [303]. One advantage of the low viscosity of fibrin is that it can be inkjet-printed. Besides fibrin alone, fibrin together with other more mechanically stable materials allows exploitation of its high bioactivity. As an example, fibrin with PCL was printed via FDM to achieve a biphasic scaffold used in cartilage tissue engineering [304,305], while fibrin gel has also been seeded into a poly(trimethylene carbonate) scaffold built by SLA [306]. Finally, fibrin scaffolds were fabricated by TPP of a master structure followed by micromolding replication via PDMS, achieving feature sizes of ~20 µm [61].

##### Biocompatibility, Biodegradability, and Bioactivity

Fibrin hydrogel encapsulated bone marrow stromal cells were inkjet printed and showed cytocompatibility of the encapsulation material [307]. Fibrin has been used as the base bioink for the printing of neural tissue as a glioblastoma model for drug screening [308]; human dental pulp stem cells for tooth tissue engineering [309]; Schwann cells for nerve tissue engineering [219,303]; human umbilical vein endothelial cells, and hMSCs for bone tissue engineering and neovascularization [310]; as well as human induced pluripotent stem cells for neurological diseases [311].

#### 4.4.3. Gelatin

##### Process and Material

Gelatin is a protein derived from collagen. It offers good biocompatibility, cell adhesion, biodegradability, and depending on the method of extraction and formulation can have different melting points, gelling temperatures, viscosity, and so on, although with poor mechanical properties. Unlike collagen it is water-soluble and can undergo sol-gel transitions that are thermo-reversible since non-specific bonds are formed when the solution is cooled, and that break open as soon as it is heated. This allows the 3D printing of gelatin. It is typically used to encapsulate cells for organ printing or mixed with other hydrogels to tune specific properties [312]. 

Besides bioprinting, photopolymerization of gelatin hydrogels for stereolithographic and light-assisted printing is well developed when compared to other natural polymers. This is due to the development of methods to modify gelatin with methacrylamide, forming GelMA, which is photocurable [313]. There are a number of detailed and up-to-date reviews on the use of GelMA for fabricating cell culturing and tissue engineering constructs [314,315,316] while broader reviews on gelatin and gelatin composites for 3D printing can be found here [312,317].

##### Structural and Mechanical Properties

Gelatin has poor mechanical properties. Therefore, to improve the bioprinting of gelatin alone, efforts have been made towards tuning the types of cross-linking in the gelatin hydrogel bioink [318], i.e., via heat-treatment or chemical post-processing, which both can preserve scaffold shape and size. Gelatin combined with other hydrogels has been used to optimize printability, improving shape retention during printing. For example, gelatin-alginate [240,319,320,321], Laponite [322], nanoclays [323], and synthetic polymers such as Pluronic F-127 [208] and PCL [324], have all been studied. Alginate dialdehyde–gelatin scaffolds were printed in the presence of a cross-linker for reaching feature sizes of ~500 µm [245], while alginate–GelMA interpenetrating networks via UV crosslinking of GelMA followed by Ca crosslinking of alginate was reported [321]. Embedded bioprinting of gelatin into a support material of agarose slurry was optimized to allow freestanding 3D structures [325].

Because GelMA is photocurable, it can be extruded as a bioink and cured simultaneously by UV light. This has allowed layer-by-layer curing of bioprinted GelMA for good stability and shape fidelity [326]. In the above study, a mixture of GelMA with gellan gum at 5%:0.5% w/v and 10%:0.2% w/v showed compressive modulus of 9 and 16 kPa, porosity of 65% to 41%, and pore size of 173 nm to 110 nm, respectively. For light-assisted fabrication alone, the preparation of GelMA and in particular the degree of substitution affects mechanical properties. Photo-patterned (including the use of TPP) GelMA scaffolds, have been produced down to ~50 µm feature size. For SLA, chondrocytes were encapsulated within a GelMA bioink and 3D structures printed with ~500 µm feature size [214,327]. The addition of PEGDA with GelMA greatly improved the SLA printing resolution to 300 µm. However, the compressive modulus changed from 0.5 MPa to 18 MPa [328]. The use of Eosin Y as a photoinitiator allowed SLA printing of GelMA using visible light [329]. Lastly, a recent development in inkjet printing of GelMA microdroplets was studied, where the use of electrostatic attraction allowed printing of ~100 µm droplets of GelMA rapidly and smoothly, with minimal damage to encapsulated cells [330].

##### Biocompatibility, Biodegradability, and Bioactivity

Gelatin is biocompatible and biodegradable. For bioprinting of gelatin alone, biocompatibility was observed over 14 days, showing non-cytotoxicity despite the presence of residues from cross-linking agents [318]. Modified furfuryl–gelatin, which is crosslinkable by visible light, was formulated together with hyaluronic acid and riboflavin and showed to be a biocompatible bioink [331]. For the preparation of GelMA, care must be taken to remove all cytotoxic by-products. In terms of cytotoxicity, the optimization of photopolymerization of GelMA with a lithium based photoinitiator showed that photoinitiator concentration was the key factor, rather than light intensity or duration of exposure [332]. Cell survival changed from 90% to 70% when the concentration of the photoinitiator was increased from 0.1 to 1 wt%.

Gelatin-alginate bioinks were used for the encapsulation and printing of mouse planta dermis [319], although cell differentiation and proliferation was low, possibly due to either shear forces during printing, cytotoxicity of the cross-linking agent, or rigidity of the composite. In contrast, printing of dental pulp stem cells for dental tissue engineering [320] and hMSCs and amniotic epithelial cells [240] using gelatin–alginate bioink showed improved cell differentiation and proliferation, indicating a role of the specific encapsulated cells or particles.

By combining GelMA with different concentrations of methacrylated hyaluronic acid and chondroitin sulphate, encapsulation of chondrocytes, bioprinting the mixture, and in situ UV curing layer-by-layer, a glycosaminoglycan-graded hydrogel was fabricated with good cell viability resulting in its own chondrocyte matrix after 28 days [333]. Chondrocytes encapsulated in a GelMA bioink and 3D printed via SLA showed chondrocyte differentiation after 14 days [214,327]. Finally, a dynamic optical projection SLA system was used to print a vascular network with encapsulated living cells [334].

#### 4.4.4. Silk

##### Process and Material

Silk fibroin is a biocompatible and non-cytotoxic protein with rigid structure, high tensile strength (360 to 530 MPa at 18 to 21% elongation), Young’s modulus between 12.4 and 17.9 GPa, and tunable degradation rate, whose sol-to-gel transition can be controlled without changes in temperature or toxic solvents [335,336,337], allowing encapsulated cells to survive extrusion. For example a spider silk based bioink was printed by robotic dispensing without any need for crosslinking additives or temperature control [338]. For even better control over shape and geometry, freeform fabrication was accomplished without post-processing by the gelation of silk within a supporting matrix of suspended Laponite nanoclay and PEG [339]. Silk fibroin scaffolds have been produced indirectly via inkjet printing a thermoplastic mold [340].

Silk fibroin can be combined with other materials to form composites with optimized properties for extrusion, mechanical strength, and bioactivity. Silk particles have also been added as additives in other material systems targeting specific functions. For example, to improve cell suspension in GelMA bioink used for DLP fabrication of 3D structures, silk fibroin particles were added to reduce viscosity [341]. There has only been one study done on methacrylation of silk fibroin, together with maleilated chitosan and the photoinitiator Darocur 2959 for UV crosslinking, where the role of the methacrylated silk fibroin particles was to modulate the compressive modulus, morphology, swelling, and sol content. The material was biocompatible with mouse articular chondrocytes, paving the way for future SLA of silk hydrogels [342].

##### Structural and Mechanical Properties

The spider silk based bioink hydrogel mentioned earlier [338] had viscoelastic properties with elastic modulus between 0.02–0.2 kPa with feature sizes of ~500 µm. Concerning the silk hydrogels fabricated within a supporting matrix of Laponite clay and PEG [339], filament widths of ~300 µm and pores of ~100 µm were printed, albeit without any encapsulated cells. For indirectly molded scaffolds [340], silk structures with features of ~700 µm were fabricated for cartilage tissue engineering applications. In this case, the presence of surface micro-pores was crucial to promote cell infiltration and adhesion, while the large micro-channels allowed cell migration towards the center of the structure as well as vascularization.

Regarding silk composites, silk fibroin was combined with gelatin and nano-hydroxyapatite for cartilage tissue engineering, achieving compressive modulus of ~1.22 MPa, printable features of ~150 µm [343], and with the possibility to tune both hydrophilicity and biodegradability.

##### Biocompatibility, Biodegradability, and Bioactivity

A spider silk based hydrogel scaffold with encapsulated human fibroblasts showed good cell adhesion, proliferation, and viability over 7 days [338]. Porcine chondrocytes showed good attachment, viability, and proliferation over 14 days [340]. For composites, a silk-gelatin bioink encapsulating calcium ions was used to print a scaffold that showed gradual calcium release that promoted osteogenic differentiation of hMSCs and enhancing mineralization processes [344]. Silk-gelatin bioinks have also been used to encapsulate hMSCs [345] and human mesenchymal progenitor cells [346]. Silk has been combined with alginate as a sacrificial hydrogel for inkjet printing of well-defined constructs with steady fibroblast proliferation after five weeks [347].

## 5. Conclusions and Future Directions

In this review we showed the advantages and the drawbacks offered by high resolution engineering approaches making use of 3D printing techniques for the realization of biomimetic cellular microenvironments. The choice of the most suitable microfabrication approach goes along with the selection of the extracellular matrix features that must be replicated. This is also directly linked to the availability of synthetic or naturally derived materials that often undergo physical or chemical modification both to tailor their biomechanical properties and to allow integration within additive manufacturing strategies. One of the major alternatives to additive manufacturing for the development of 3D microphysiological systems is represented nowadays by scaffold-free approaches making use of cellular self-assembly leading to the formation of large scale constructs called spheroids [348] and organoids [349]. Although being able to reproduce fundamental features of living tissues, these architectures, that often grow in an uncontrolled way, can suffer of early necrosis and batch-to-batch variability. To overcome these limitations, the combination of standardized additive manufactured niches and preformed multi-cellular constructs [350] would probably be the next challenge for obtaining true biomimetic models.

## Figures and Tables

**Table 1:** Summary of results for the fabrication of thermoplastic and soft polymer 3D microenvironments for cell culturing (in brackets we highlighted the paragraph number to which the materials/fabrication approaches are related).

	SLS (3.1)	FDM (3.2)	Extrusion Bioprinting (3.3)	Light-Assisted (3.5)	Applications
PCL (4.1.1)	Porosity 40–80% [41]; Compressive E: 10–60 MPa, str.: 0.6–10 MPa [38,39,40,41]; Inclusion of β-TCP [36,88,89,90], HA [91,92,93]	Feature size 160 µm, tensile E: 4–77 MPa, str.: 0.4–3.6 MPa [79]; Inclusion of glass [95], elastomers [96], β-TCP [90], PU [97], PLA [98,99], cellulose [101]	Feature size 200 µm [80,81,82,83]; Inclusion of PEGDMA resulting in E: 65 MPa, hardness of 6 MPa [83]	Feature size 50 µm (DLP) [84]; Feature size 1–10 µm (TPP) [85,86]; Tensile E: 3.3–6.9 MPa [84,86]; Compressive E: 0.27–4 MPa [87]	Bone tissue engineering [110,116,119]; Chondrocytes [118]; hMSCs [119]
PPF (4.1.2)		Pore size range of 0.1–1 mm [122]		Feature size 150 µm [124]; Compressive E: 4 MPa–4 GPa [131]; Inclusion of b-TCP [123], HA [127], TiO2 [128], CNTs [129]	Human chondrocytes for cartilage tissue engineering [124,135]; Neurotrophin-3 for neurons and axons [136]; Adipose stem cell adhesion for tissue regeneration [137]; rat bone-marrow stromal cells [140]; hMSCs [141]
PLA (4.1.3)	[142]; Inclusion of CaCO_3_ with bending strength: 75 MPa [34]	Feature size 100 µm [143]; Inclusion of HA, compressive E: 4.7–9.8 MPa, str.: 0.29–0.44 MPa [154], GO [143]		Feature size 1 mm (SLA) [150]; Feature size 20 µm (TPP) [61]	Human fetal osteoblasts [145]; human bone-marrow stromal cells [143]; Schwann cells [61]; osteoconductivity [147,158]; bacterial cell colonies [142]
PEEK (4.1.4)	Inclusion of PGA and PLLA for better degradability [163,164,165]	Elastic E: 2.5–4.1 GPa, tensile str.: 23–84 MPa [157,158,161]	Inclusion of fenchone, tensile E: 2 GPa, str.: 13 MPa [159]		hMSCs [166]
PEG (4.2.1)			Inclusion of nanosilicates [172]; Feature size 500 µm, E: 50 kPa [174]	Feature size 25 µm (SLA) [173]; Feature size 5 µm (TPP) [177,178,179]; Inclusion of silk and melanin, storage E: 1–2.5 kPa [134]	Anti-bacterial [183]; hMSCs [174]
PDMS (4.2.2)			Feature size 250 µm [194]	Feature size 250 µm (SLA) [195]; Feature size <1 µm (TPP) [196]; E: 0.4–1.7 MPa [199]; E: 0.05–1 MPa [200]	Anti-bacterial [47]

**Table 2:** Summary of results for the fabrication of polysaccharide and protein based 3D microenvironments for cell culturing (in brackets we highlighted the paragraph number to which the materials/fabrication approaches are related).

	Extrusion bioprinting (3.3)	Light-Assisted (3.5)	Applications
Hyaluronic acid (4.3.1)	[207,208,209]; Inclusion of GelMA, feature size 500 µm [210]; Cryogel E: 2–2.5 kPa [211]; Post-curing via UV, E: 1.3–10.6 kPa [213]	Feature size 300 µm (SLA) [214]; Compressive E: 780 kPa [215]	Cartilage tissue engineering and human adipose stem cells [215]; stromal cell elongation and drug screening [209]; retinal cell culturing [216]; hMSCs [217]; human adipose progenitor and stromal cells [211]; Schwann cells [219]
Chitosan (4.3.2)	[222,223]; Feature size 50 µm [224]	[225,226]; Feature size 50 µm (SLA), E: 160–680 kPa [173]; Feature size 400 nm (TPP) [228]; Inclusion of HA [229]	Anti-bacterial [230,231]; wound dressings [232]; skin tissue engineering [231]; bone tissue engineering [229]; pluripotent stem cells for neural tissue engineering [233]; articular cartilage tissue engineering [234]; skin constructs [235]
Alginate (4.3.3)	Inclusion of NiCu [237], e-polylysine [238], carrageenan [239], gelatin [240,241,242], cellulose [243,244], PVA [252], TiO_2_ [253], β-TCP [253]; Feature size 150 µm, E: 280 kPa [240]		Myoblasts [242]; endothelial cells [259]; E.coli [260], growth factor [250], human adipose stem cells [248]; human induced pluripotent stem cells [261]; chondrocytes [243,244,251]; Schwann cells [265]
Cellulose (4.3.4)	Mixed with alginate [244,266,267,268,269,270,271,272]; As reinforcement material, E: 2.5–22.5 kPa [281]	Feature size 500 µm [280]; Tensile E: 0.67–0.63 GPa [282]	Chondrocytes for cartilage tissue engineering [244,269,271]; human induced pluripotent stem cells, bone-marrow hMSCs [272]; pancreatic cancer cells [268]; fibroblast and hepatoma cells [270]; NIH 3T3 cells [274]
Collagen (4.4.1)	Freeform reversible embedding of suspended hydrogels [288]; Compressive E: 10–30 kPa [294]; Inclusion of genipin, feature size 400 µm, compressive E: 17 kPa to 1.4 MPa [295]	Indirect coating of collagen onto TPP scaffold, feature size 30 µm [290]	Osteoblast cells, human adipose stem cells [295]; tissue spheroids [296]; keratinocytes and fibroblasts [297], hMSCs [298]; human corneal epithelial cells [257]; osteocytes [300,301,302]; 3D liver microenvironments [209]
Fibrin (4.4.2)	Mixed with PVA, feature size 100 µm [303]	Indirect methods of coating [306], micro-molding with feature size 20 µm [61]	Bone marrow stromal cells [307]; neural tissue [308]; dental pulp stem cells [309]; Schwann cells [219,303]; human umbilical vein endothelial cells, hMSCs [310]
Gelatin (4.4.3)	Inclusion of alginate [240,319,320,321], Laponite [322], nanoclays [323], Pluronic F-127 [208], PCL [324]; Feature size 500 µm [245]	[313,314,315,316]; Feature size 300 µm (SLA), Compressive E: 0.5–18 MPa [328]	Mouse planta dermis [319]; dental pulp stem cells [320]; hMSCs and amniotic epithelial cells [240]; chondrocytes [214,327]
Silk (4.4.4)	[335,336,337,338]; Feature size 500 µm, elastic E: 0.02–0.2 kPa [338]; Inclusion of HA, feature size 150 µm, compressive E: 1.22 MPa [343]	Compressive E: 0.32 MPa [342]	Mouse articular chondrocytes [342]; human fibroblasts [338]; porcine chondrocytes [340]; hMSCs [344,345]; human mesenchymal progenitor cells [346]

## Abbreviations

AM	Additive manufacturing
CNT	Carbon nanotubes
DEF	Diethyl fumarate
DLP	Digital light processing
ECM	Extracellular matrix
FDM	Fused deposition modeling
FFF	Fused filament fabrication
GelMA	Gelatin methacrylate
GO	Graphene oxide
HA	Hydroxyapatite
hMSCs	Human mesenchymal stem cells
PCL	Polycaprolactone
PDMS	Polydimethylsiloxane
PEEK	Polyether-ether-ketone
PEG4A	PEG-tetraacrylate
PEGDA	Poly(ethylene glycol) diacrylate
PEGDMA	Polyethylene glycol dimethyl-acrylate
PEGDTT	PEG-dithiothreitol
PEGMEA	Poly(ethylene glycol) methyl ether acrylate
PEGNB	PEG norbornene
PGA	Polyglycolic acid
pHEMA	Poly(2-hydroxyethyl methacrylate)
PLA	Polylactic acid
PLGA	Poly(lactic-*co*-glycolic acid)
PLLA	Poly-L-lactide
PMMA	Poly(methyl methacrylate)
PPF	Polypropylene fumarate
PVA	Poly(vinyl alcohol)
RGD	Arginine-glycine-aspartic acid
SLA	Stereolithography
SLS	Selective laser sintering
TCP	Tricalcium phosphate
TPP	Two-photon polymerization
